# Freezing of Enkephalinergic Functions by Multiple Noxious Foci: A Source of Pain Sensitization?

**DOI:** 10.1371/journal.pone.0006874

**Published:** 2009-09-03

**Authors:** François Cesselin, Sylvie Bourgoin, Annie Mauborgne, Michel Hamon, Daniel Le Bars

**Affiliations:** 1 Team “Pain”, INSERM UMRS 975, CNRS UMR 7225, Paris, France; 2 Université Pierre et Marie Curie, Faculté de Médecine UPMC, Paris, France; 3 INSERM UMR 677, Paris, France; INSERM U862, France

## Abstract

**Background:**

The functional significance of proenkephalin systems in processing pain remains an open question and indeed is puzzling. For example, a noxious mechanical stimulus does not alter the release of Met-enkephalin-like material (MELM) from segments of the spinal cord related to the stimulated area of the body, but does increase its release from other segments.

**Methodology/Principal Findings:**

Here we show that, in the rat, a noxious mechanical stimulus applied to either the right or the left hind paw elicits a marked increase of MELM release during perifusion of either the whole spinal cord or the cervico-trigeminal area. However, these stimulatory effects were not additive and indeed, disappeared completely when the right and left paws were stimulated simultaneously.

**Conclusion/Significance:**

We have concluded that in addition to the concept of a diffuse control of the transmission of nociceptive signals through the dorsal horn, there is a diffuse control of the modulation of this transmission. The “freezing” of Met-enkephalinergic functions represents a potential source of central sensitization in the spinal cord, notably in clinical situations involving multiple painful foci, e.g. cancer with metastases, poly-traumatism or rheumatoid arthritis.

## Introduction

The sites of the first synapses in pain pathways, namely the dorsal horn of the spinal cord and its trigeminal homologue in the brainstem, the nucleus caudalis, are amongst the main loci for the integrative processing of nociceptive information. These areas are rich in opioid receptors and in neurons containing proenkephalin-A and B derivatives [1]. Although electrophysiological, biochemical, behavioral and clinical studies have all illustrated the potential of spinal opioidergic systems to control the transmission of pain signals, the question still remains open as to the functional significance of such systems.

Direct measurements of the release of endogenous opioids from the spinal cord of the rat and the cat have shown that noxious stimuli can trigger activity in spinal opioidergic systems [1]. Noxious pinches increased the spinal release of Met-enkephalin-like material (MELM). However, noxious mechanical stimuli do not alter the release of MELM from neural segments related to the stimulated area of the body, but increase its release from other segments [2]. Together with lesion experiments [2], these findings suggested the simultaneous triggering of excitatory and inhibitory processes by noxious mechanical stimuli, the former triggering the neuronal firing of met-enkephalinergic neurons through a spino-bulbo-spinal loop and the latter blocking such a firing at a segmental level. This theoretical possibility opens up a large number of hypotheses involving interneuronal networks.

Since many areas of the body could be the trigger for the release of MELM from a given spinal segment, the aim of the present study was to determine the type of interaction between stimuli-induced MELM release triggered from several distant areas. We have chosen the hind paws for convenience. Either the whole spinal cord or the cervico-trigeminal area was perifused. The former was chosen for a general view of the spinal release, and the latter for the investigation of release in parts of the spinal cord, which are unambiguously distant from where afferent projections from the hind paws terminate. We show here that a noxious mechanical stimulus applied either to the right or the left hind paw elicited a marked increase of MELM release during perifusion of either the whole spinal cord or the cervico-trigeminal area. However, when the right and left paws were stimulated simultaneously, not only were these stimulatory effects not additive but they completely disappeared.

## Results

**Figure 1:**
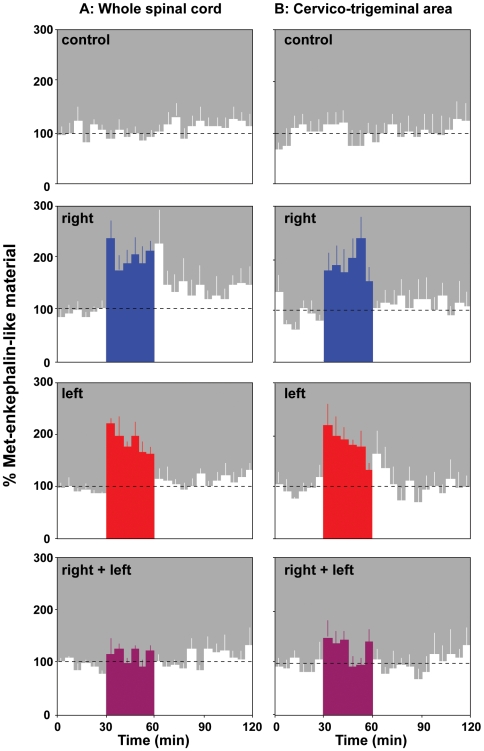
Effects of mechanical stimuli on the release of Met-enkephalin-like material (MELM) during the perifusion of the whole spinal cord (A) or the cervico-trigeminal area (B). Following a 30-minutes control period (spontaneous release: A = 2.4±0.6 and B = 1.9±0.2 pg/5 min), calibrated noxious pinches (10 N/cm^2^, 10 s duration, 3 times per min) were applied repetitively for 30 minutes (black areas) in four groups of animals in a Latin square experimental design. From top to bottom: no stimulation (controls), stimulation of the right hind paw, stimulation of the left hind paw, stimulation of both the right and the left hind paws. Note that MELM release increased markedly when pinches were applied to either hind paw. No effect was seen when both hind paws were stimulated simultaneously. Results are expressed in terms of percentage of the mean basal value observed during the control period. ANOVA and *post hoc* PLSD Fisher tests indicated highly significant effects of both individual factors of variation and their interactions (see table S1), respectively.

In halothane anesthetized rats, either the whole spinal cord or the cervico-trigeminal area were perifused with an artificial cerebro-spinal fluid and fractions were collected where the spontaneous release of MELM corresponded to 2.4±0.6 and 1.9±0.2 pg per 5 minutes, respectively. Calibrated noxious pinches were applied for 30 min either to the right, the left or both hind paws. In whole spinal cord perifusates (Fig. 1A), MELM release was found to increase markedly when pinches were applied to either hind paw (mean increases: by 105.3±20.9 and 88.5±27.4% for the right and left hind paws, respectively) and then rapidly returned to the control values after the cessation of stimulation. When both hind paws were stimulated simultaneously, no effect was seen. Very similar results were seen during perifusion of the cervico-trigeminal area (Fig. 1B; mean increases: by 90.9±17.7 and 88.9±14.1% for the right and left hind paws, respectively). Again, when the right and left paws were stimulated simultaneously, the stimulatory effect disappeared. During perifusion of both the whole spinal cord and the cervico-trigeminal area, the interaction between the two factors of variation was found to be highly significant indeed (F_1-27_ = 24.33 and F_1-22_ = 30.17, respectively, p<0.0001; Table S1).

## Discussion

**Figure 2:**
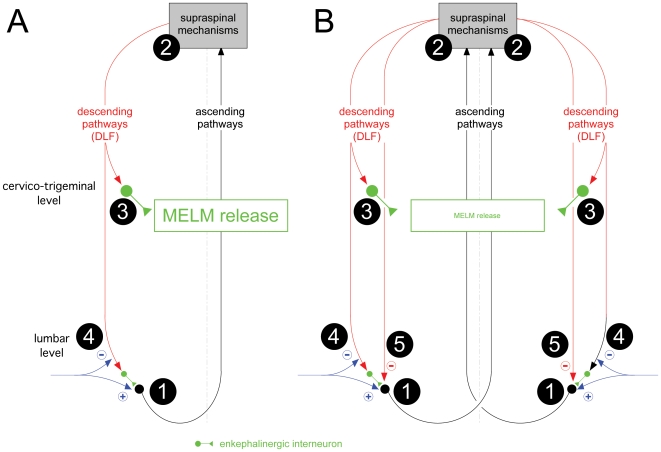
Hypothetical pathways regulating MELM release elicited by noxious mechanical stimuli. A. Stimulus applied on a single area. The peripheral input (blue) activates dorsal horn neurons (1) that project to the brain. Descending controls are triggered (2) that produce a series of influences on dorsal horn neurons through the DLF. One of these triggers a diffuse release of Met-enkephalin (3). However, such a release is prevented by the blockade of afferent inputs at the segmental level (4) (see references 2 & 3). B. Stimuli applied to two body areas (e.g. right and left paws). Identical processes are triggered from each stimulation site (1, 2, 3, 4) but the power of DNIC is strong enough to produce a functional block of firing as early as the dorsal horn (5).

It was confirmed that noxious mechanical stimuli increased the release of MELM heterosegmentally in the rat spinal cord [2]. Since bilateral lesions of the dorsolateral funiculus (DLF) completely blocked such stimulatory effects [2], they must be mediated via an ascending-descending pathway (Fig. 2A). In addition, the similarity of results observed during whole spinal cord or cervico-trigeminal perifusion, confirmed that segmental and/or propriospinal mechanisms were not involved in these processes.

However, as shown here, such increases were blocked when multiple noxious foci were involved. Several hypotheses could explain the negative interaction seen following stimulation of the two hind paws. First, it could be hypothesized that a too large release of MELM provided by stimulation of both hind paws triggered a presynaptic inhibition of the peptide release. Indeed, a feedback inhibition of MELM release from the rat spinal cord has been demonstrated [3]. However, this interpretation appears to be very unlikely. When the extracellular levels of enkephalins are elevated by the blockade of their enzymatic degradation (e.g. by kelatorphan), a noxious mechanical stimulation is as efficient as in the absence of the peptidase inhibitor for producing a sharp increase in MELM outflow: absolute levels of released MELM were higher, but the relative effect of the noxious stimulation was identical [4]. One could also hypothesize that the spinal release of Met-enkephalin is highly modulated by supraspinal controls, which themselves are activated by the noxious foci and involve an ascending/descending drive to the enkephalinergic interneurons (Fig. 2B). Many structures in the brain have been reported to be the source of a descending inhibitory control of dorsal horn neurons involved in the spinal transmission of nociceptive information [5]–[7]. On the basis of anatomical links between ascending pain pathways and some of these structures, the triggering of descending inhibition by noxious stimuli has been postulated (5–7). There are three main possibilities regarding such structures.

It has been shown that many lamina 1, noxious-specific neurons in the dorsal horn send axons through the DLF towards the parabrachial area [8], [9], which can be a source of descending inhibition [5]–[7]. On the other hand, the rostral ventromedial medulla sends many axons through the DLF towards all levels of the dorsal horn [10] and is the source of powerful inhibitions of dorsal horn neurons involved in the processing of nociceptive information [5]–[7]. However, negative interactions between activities resulting from stimuli applied to distant parts of the body were not described in studies involving recordings of parabrachial or rostral ventromedial medulla neurons. To the best of our knowledge, only one brain structure has been reported to contain neurons in which strong negative interactions have been observed as a result of noxious stimuli being applied simultaneously to two different areas of the body - namely the subnucleus reticularis dorsalis (SRD) in the caudal medulla. The SRD contains neurons with characteristics that suggest they play a key role in the processing of nociceptive information [11]. Indeed, they are preferentially or exclusively activated by nociceptive stimuli from “whole-body” receptive fields, they encode the intensity of cutaneous and visceral stimulation within noxious ranges and they are excited exclusively by activity in cutaneous Aδ- or Aδ- and C-fibres. In addition, they send descending projections through the DLF that terminate in the dorsal horn at all rostro-caudal levels of the spinal cord. The firing of SRD neurons during simultaneous noxious stimulation of the two hind paws was found to be much less than the firing elicited by stimulating either one or the other paw [12]. Since such effects disappeared in animals with bilateral DLF lesions [12], it follows that the SRD is a good candidate as a brain structure involved in the effects described in the present study.

At the spinal level, supraspinally mediated inhibitory controls triggered by noxious stimuli have been described as “Diffuse Noxious Inhibitory Controls” (DNIC). In the rat [13]–[21], the mouse [22], the cat [23], [24] and the monkey [25], [26], most wide-dynamic-range and some nociceptive-specific neurons in the dorsal horn are strongly inhibited by a noxious stimulus applied outside their excitatory receptive fields. Such effects are not organized somatotopically but apply to the whole body. For example, a neuronal response to a pinch applied to a hind paw is inhibited by a pinch applied to any other part of the body, including the controlateral hind paw. Interestingly, systemic naloxone (an opioid receptor antagonist) reduces DNIC in both rats [27] and man [28]. Thus, it is very likely that the enhanced release of MELM observed in the present study following stimulation of only one hind paw might participate in DNIC. In addition, several lines of evidence lead us to believe that the interactions between noxious inputs described herein could also be sustained by DNIC. Most particularly, there are several features which are shared by the two phenomena, notably the fact that DNIC are elicited specifically by any heterotopic noxious stimuli, have no apparent somatotopic organization even on a very large scale, and disappear following DLF lesions [29]. Interestingly, lesions of the SRD strongly reduced DNIC [30].

In summary, DNIC have all the requirements of diffuse inhibitory mechanisms necessary to explain the negative interactions between noxious inputs observed during measurements of spinal MELM release and described herein. Although this assertion is supported by converging arguments, none of them are sufficient for a definite conclusion to be put forward.

The present study suggests that DNIC indirectly affect the activities of spinal met-enkephalinergic neurons. DNIC are triggered by a large variety of stimuli including traditional manual acupuncture (lifting, thrusting and rotating the needle in a clockwise and anti-clockwise fashion at 2–4 Hz). We observed that such a stimulus, which is known to elicit widespread extrasegmental antinociceptive effects [31], is able, under identical experimental conditions: (1) to activate SRD neurons [32]; (2) to inhibit the activities of dorsal horn wide-dynamic-range neurons [33]; and (3) to activate spinal met-enkephalinergic neurons [34].

Our results extend the notion of diffuse controls triggered by noxious inputs from affecting the transmission of nociceptive signals (i.e. DNIC) to also affecting the modulation of this transmission. Regarding clinical pain, our results strongly suggest that the occurrence of multiple painful foci, as seen for example with cancer pain with metastases or poly-traumatisms, could result in a “freezing” of the Met-enkephalinergic functions in the spinal cord. We have only considered here, short-lasting noxious stimuli; the evolution of such a freezing during the development of chronic pain needs to be investigated carefully. There are elements to suggest this is the case with rheumatoid arthritis. Polyarthritis elicited by the immunogenic complete Freund's adjuvant is a validated model of human rheumatoid arthritis [35] that produces behavioral disturbances related to spontaneous pain [36]. Increased basal tissue concentration of MELM was seen in the spinal cord of these animals [37] and this was associated with a clear reduction of release [38]. The “freezing” of Met-enkephalinergic functions could therefore be an important source of central sensitization in the spinal cord. Indeed, the inhibitory role of Met-enkephalin is a classical notion that confers to this molecule, the physiological potential of reducing pain. Blocking its release would tilt the beam of the balance in the opposite direction, an exacerbation of pain, which would be felt more intensely than normal.

## Materials and Methods

Male Sprague-Dawley rats weighing 320–380 g were kept under controlled environmental conditions (22°C, 12 h alternate light-dark cycles, 50% humidity, food and water *ad libitum*) for at least 7 days before being used in the experiments. The National Institute of Health's “Guide for the care and use of Laboratory animals”, the European Communities Council Directive 86/609/EEC, and the Committee for Research and Ethical Issues of the International Association for the Study of Pain (IASP) on ethical standards for investigations of experimental pain in animals were followed. The surgical procedures were performed under deep anesthesia (2% halothane in a nitrous oxide-oxygen mixture, 66/33, v/v). After tracheal cannulation, allowing artificial ventilation, and insertion of a catheter in the right inner jugular vein, the animal was immobilized in a ventroflexed position using a Horsley-Clarke apparatus. The rat was artificially ventilated, the rate and the volume being adjusted to maintain a normal acid-base equilibrium [39]. All along the experiment, vital parameters were controlled.

The method of perifusion of the whole intrathecal space was adapted from that described by Yaksh and Tyce [40]. A transverse incision was made over the external occipital crest and on the midline overlying the cisterna magna. Muscles were drawn aside from the skull and atlas, and the occipital-atlantoidal membrane was carefully retracted from the cisterna dura. A small incision of the dura and the arachnoid was made over the obex. A nylon inflow catheter (Polyethylene tubing PE10; 0.28 mm inner diameter, 0.61 mm outer diameter) was then carefully inserted and conveyed 85 mm into the subarachnoid space to the lumbar region. An outflow catheter (same tubing) was inserted parallel to the former, with its extremity overlying the lower medulla.

For the perifusion of the cervico-trigeminal area, the inflow catheter was inserted to a point 15 mm caudal to the obex (i.e. to the C4–C5 segments), so that the perifused zone corresponded to the trigeminal and cervical (C1–C3) areas.

Following the surgical procedures, the rats were paralyzed by slow i.v. infusion of gallamine triethiodide, and the level of halothane was lowered to 0.9% for the remainder of the experiment to achieve an adequate level of anesthesia for ethical considerations while not excessively depressing neuronal responses to noxious stimuli [41]. The spinal cord was then perifused with an artificial cerebrospinal fluid (in mM: NaCl 126.5; NaHCO_3_ 27.5; KCl 2.4; KH_2_PO_4_ 0.5; CaCl_2_ 1.1; MgCl_2_ 0.85; Na_2_SO_4_ 0.5; glucose 5.9) adjusted to pH 7.3 by bubbling with an O_2_/CO_2_ mixture (95: 5, v/v) and maintained at 37°C at the output of the inflow catheter. The flow rate was 0.1 ml/min. Perifusion for 30–45 min before collecting the first samples allowed the release of MELM to be stable for at least the following 180 min, corresponding to the whole perifusion [42]. Thereafter 0.5 ml fractions (corresponding to 5 min) were collected on dry ice and stored frozen at −30°C until the measurement of their MELM content. Six fractions (corresponding to 30 min of perifusion) were collected before any treatment was applied. MELM was measured in the perifusates using a radioimmunoassay procedure. The antiserum used in the present study was raised in a rabbit by injections of Met-enkephalin coupled to ovalbumin with 1-ethyl-3(3-dimethyl-aminopropyl)carbodiimide. The reaction was performed in the presence of [^3^H]met-enkephalin. After extensive dialysis treatment against 0.9% NaCl, the conjugate was estimated to contain 6 enkephalin residues per molecule of ovalbumin. An aliquots corresponding to 1.5 mg of the conjugate was emulsified in complete Freunds's adjuvant (final volume: 1.2 ml) and injected intradermally into the axillar and crural regions of a white male rabbit (2.5 kg, HY/CR strain). It was bled 3 weeks later and repeatedly boosted with 0.75 mg of the antigen conjugate approximately every month. The collected serum was heated (30 min a 56°C), then mixed with an equal volume of glycerol and kept at −30°C. Results presented in this paper were obtained with the serum obtained 1 month after the 5^th^ booster injection. MELM was measured in the superfusates using a slight modification of the procedure already described [43]. Briefly, each 0.5 ml fraction was thawed and mixed with 0.05 ml of 0.025 M Tris-Hcl, pH 7.6, containing 0.5 mg/ml bovine serum albumin and 0.05 ml of the antiserum (1∶10,000 final dilution). Standard curves were obtained under the same conditions using 0.5 ml of artificial cerebrospinal fluid. After 48 h at 4°C, 0.05 ml of a [Tyrosyl-^125^I]met-enkephalin solution (corresponding to 2000–3000 counts/min) was added and the incubation continued for 24 h. The assay was stopped by adding 1 ml of a charcoal suspension (1 mg/ml) in 0.025 M Tris-Hcl, pH 7.6, containing 0.1 mg/ml of Dextran T70. After centrifugation (6,000 g, 15 min, 4°C), [Tyrosyl-^125^I]met-enkephalin bound to the antibodies was measured in the supernatant using a gamma counter. Under these conditions, as little as 0.5 pg of ME could be quantitatively estimated in 0.5 ml of perifusate. Analysis of the binding characteristics of the antiserum indicated that, among all the possible derivatives of proenkephalin-A and -B and pro-opiomelanocortin, only ME-Arg^6^, ME-Arg^6^-Phe^7^ and ME-Lys^6^ interfered in the assay (36%, 19% and 3% cross-reactivity, respectively, as compared to 100% with ME). In addition, the sulphoxide derivative of ME cross-reacted at 360%. In contrast, cholecystokinins, substance P and somatostatin were inactive (less than 0.01% cross-reactivity) [42].

The rate of spontaneous release of MELM was stable, allowing the mean MELM content of the 6 fractions preceding the application of noxious mechanical stimuli (noxious pinches of 10 seconds duration applied 3 times per minute for 30 min to the hindpaw using calibrated forceps) to be taken as the control value (100%), and any subsequent changes in MELM release to be expressed as percentages of this value.

The means±S.E.M. were calculated from such data obtained in 6–9 rats. Statistical analyses were carried out using ANOVA followed by *Post hoc* PLSD Fisher tests, when needed.

## Supporting Information

Table S1 (0.03 MB PDF)
