# Supplementary material for: Freezing of Enkephalinergic Functions by Multiple Noxious Foci: A Source of Pain Sensitization?
Source: PLoS One. 2009 Sep 3;4(9):e6874. doi: 10.1371/journal.pone.0006874 (PMC2731161; doi:10.1371/journal.pone.0006874)
Supplement: Table S1 — (0.03 MB PDF) [file pone.0006874.s001.pdf]

Freezing of Enkephalinergic Functions by Multiple Noxious Foci: a Source of Pain Sensitization?

François Cesselin, Sylvie Bourgoin, Annie Mauborgne, Michel Hamon & Daniel Le Bars

SUPPLEMENTARY INFORMATION

(A) Whole spinal cord

Source of variation	Sums of squares	Degrees of freedom	Mean squares	F_{1-27}	p
Right paw (R)	1624.5	1	1624.5	0.596	0.4469
Left paw (L)	41.2	1	41.2	0.015	0.9031
Interaction (R x L)	66351.4	1	66351.4	24.33	< 0.0001
Error	73937.5	27	2727.3		
Total	141954.6	30			

(B) Cervico-trigeminal area

Source of variation	Sums of squares	Degrees of freedom	Mean squares	F_{1-27}	p
Right paw (R)	1386.0	1	1386.0	1.18	0.29
Left paw (L)	1019.2	1	1019.2	0.87	0.36
Interaction (R x L)	35512.3	1	35512.3	30.17	< 0.0001
Error	25894.3	22	1177.0		
Total	63811.8	25			

Supplementary Table 1. Analysis of variance of the effect of noxious pinches applied to the right (factor R) and left (factor L) hind paws on the MELM release in the whole spinal cord (A) and the cervico-trigeminal area (B). Note the very high significance of the interaction between the two factors of variation in both the whole spinal cord and the cervico-trigeminal area.
